# Temporal Colonic Gene Expression Profiling in the Recurrent Colitis Model Identifies Early and Chronic Inflammatory Processes

**DOI:** 10.1371/journal.pone.0050388

**Published:** 2012-11-30

**Authors:** Bas Kremer, Rob Mariman, Marjan van Erk, Tonny Lagerweij, Lex Nagelkerken

**Affiliations:** 1 Department of Metabolic Health Research, TNO, Leiden, The Netherlands; 2 Department of Immunohematology and Bloodtransfusion, Leiden University Medical Centrum, Leiden, The Netherlands; 3 Department of Microbiology and Systems Biology, TNO, Zeist, The Netherlands; Institut Pasteur de Lille, France

## Abstract

The recurrent TNBS-colitis model in BALB/c mice has been proposed as a model of Inflammatory Bowel Disease with a shifting pattern of local cytokines with the expression of Th1 cytokines during the early phase, Th17 cytokines during the intermediate phase and Th2 cytokines during late fibrotic stages. In this study, we evaluated the development of pathology in time–in conjunction with genome-wide gene expression in the colons–in response to three weekly intrarectal instillations of TNBS. During this time-frame mice develop colitis with extensive cellular infiltration of (sub)mucosa and mildly to moderately affected crypt architecture. These pathological processes were sensitive to local treatment with budesonide. Gene expression profiling confirmed an acute phase response after each intrarectal TNBS-challenge. In addition, a chronic inflammatory process developed over time particularly evident from a gradual increase in expression of mast cell related genes. The changes in pathological hallmarks were consistent with a temporal expression of mRNA encoding a selection of chemokines. In conclusion, the early stages of the recurrent TNBS-colitis model reflect several aspects of inflammatory bowel disease which are sensitive to immunomodulation.

## Introduction

Inflammatory Bowel Diseases (IBD), with Ulcerative Colitis (UC) and Crohn’s Disease (CD) as major entities, are chronic inflammatory disorders of the gastrointestinal tract, affecting an estimated 3.6 million people in Europe and the US [1]. IBD may be chronic or relapsing in nature, possibly due to a process of inflammation following an exaggerated immune response against enteric microorganisms. These processes affect intestinal function leading to diarrhea, cramping, and abdominal pain. Although considerable progress has been achieved with regard to the multifactorial nature of IBD [2], current therapies show limited efficacy [3].

Although there is a clear medical need to develop novel anti-inflammatory therapy for IBD, the evaluation of novel therapeutic strategies is hampered by the availability of suitable preclinical efficacy models. Most frequently, acute colitis models such as dextrane sodium sulphate (DSS) and the 2,4,6-trinitrobenzene sulfonic acid (TNBS) induced colitis are used [4], [5]. These models are characterized by severe acute inflammation of the colon representing histopathological aspects that reflect human IBD. Whereas the DSS-colitis model involves activation of the innate immune system in particular, the TNBS model was shown to depend on a local Th-1 biased response [6]. The acute chemically induced IBD models have been shown of great value for a better understanding of acute inflammatory disease processes in IBD. In addition, efficacies of a limited number of relevant treatments have been reported in these models [7]–[9].

As an alternative to chemically induced colitis, several models helped to gain valuable insight into the underlying pathology of IBD, such as the model based on transfer of CD45RB^Hi^ CD4^+^ T cell to immunodeficient recipients or the development of colitis in IL-10 deficient mice [10], [11]. These models put emphasis on the role of the intestinal microbiota eliciting a chronic inflammatory response in the gut. However, because these models are performed in an immunodeficient environment with disturbed immunoregulatory circuits they are not always suitable for the evaluation of novel therapeutic strategies.

In recent years, several groups have demonstrated that repeated intrarectal administrations of low-dose TNBS to mice and rats result in chronic colitis [12], [13]. This colitis model has primarily been used to study fibrotic processes as a consequence of chronic intestinal inflammation [14], [15]. Recently, we have implemented a modified protocol of the chronic TNBS colitis model with reduced duration, in which treatment with probiotics was shown to prevent the onset of chronic colitis and associated clinical symptoms, suggesting sensitivity to immunomodulation [16].

In this report, we characterize the processes underlying the development of pathology in this model by temporal gene expression profiling in the colon with the aim to further substantiate the value of this model for both mechanistic and pre-clinical efficacy studies. We show that the recurrent TNBS-induced colitis model combines histopathological features with aspects of active inflammation and chronic processes of cellular infiltration, angiogenesis and tissue remodeling, which are all relevant to human IBD.

**Figure 1:**
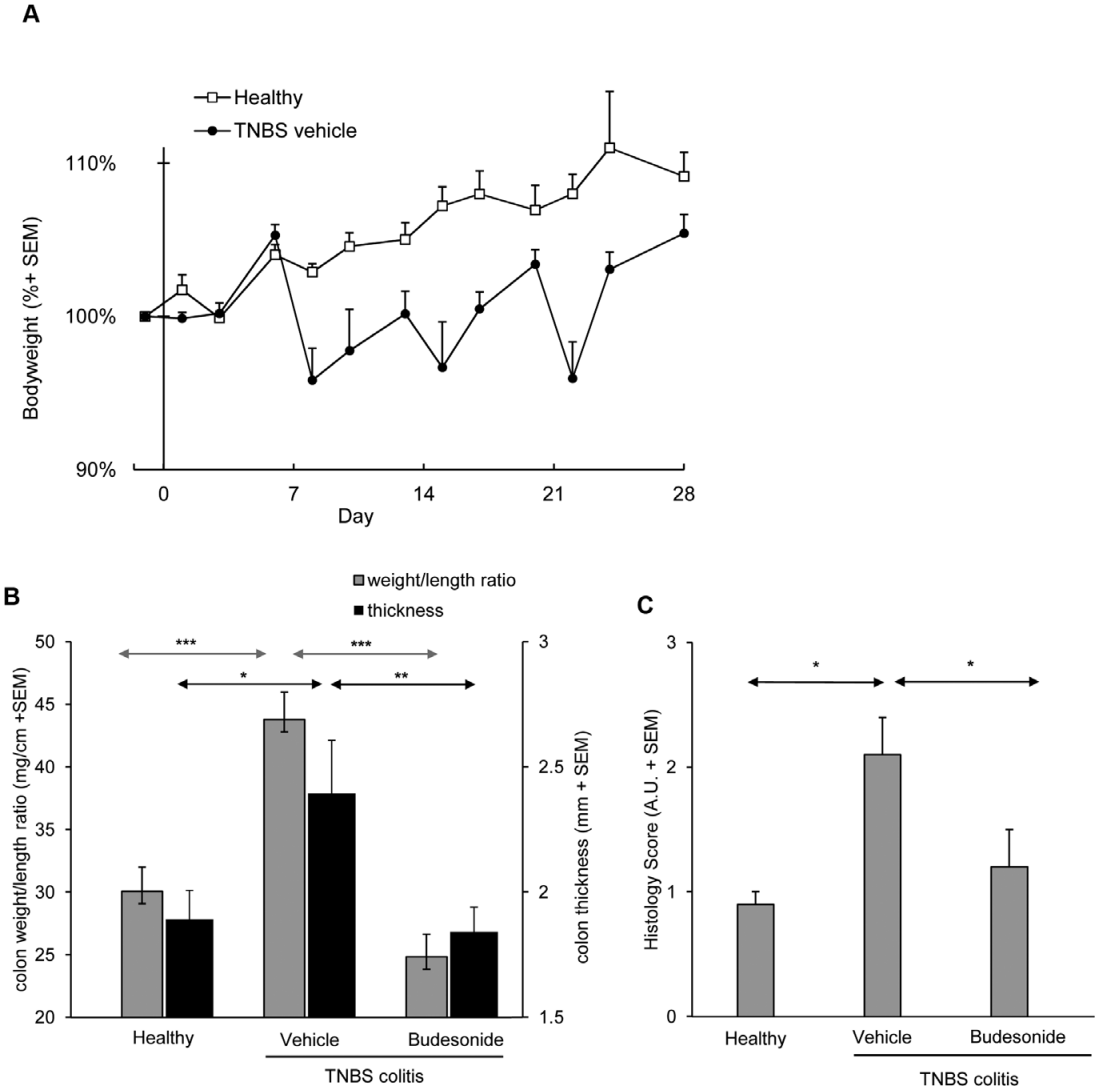
Disease characteristics of the recurrent TNBS model and efficacy of budesonide treatment. The development of colitis induction is evident from periods of loss of body weight following each rectal instillation of TNBS (Figure 1A; AUC p<0.005). At end-point, inflamed colons show significantly increased weight/length ratios and colon thickness (Figure 1B); both aspects are suppressed by budesonide. TNBS colitis induction is associated with a significantly increased histological score (Figure 1C); this score is significantly lower in budesonide treated animals. Bars represent group mean values of 10–12 mice/group, error bars represent SEM. Mann-Whitney U-test; *p<0.05, **p<0.01, ***p<0.001.

**Figure 2:**
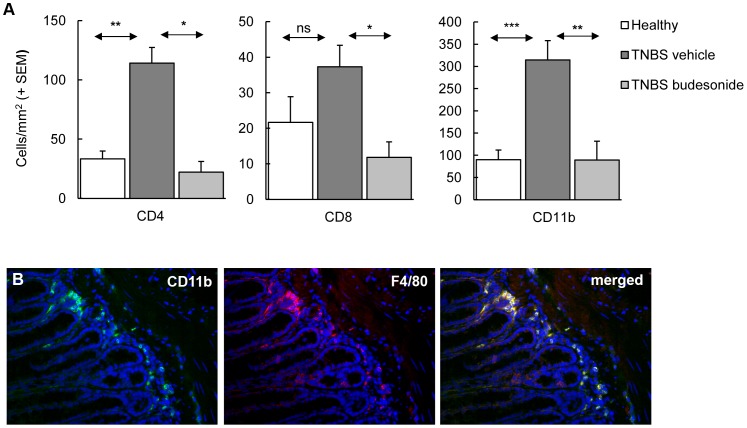
Colitis induction is associated with increased numbers of innate and adaptive immune cells. The composition of the cellular infiltrate and the effect of budesonide treatment were analyzed by immunohistochemistry. (**A**) Enumeration of the cells showed significantly increased cell numbers per mm^2^ tissue due to TNBS colitis induction. Budesonide treatment significantly reduced the number of these infiltrating cells. Bars represent group mean values of 10–12 mice/group, error bars represent SEM. Mann-Whitney U-test; *p<0.05, **p<0.01, ***p<0.001. (**B**) Double staining of CD11b+ and F480+ cells using fluorescence microscopy confirmed co-expression of these two cell surface markers.

## Results

### Recurrent TNBS-colitis: a Model with Low Mortality Sensitive to Intrarectal Budesonide

In this study, we evaluated early stages of the recurrent TNBS colitis model and confined ourselves to 4 weeks of follow-up after skin sensitization and three weekly intrarectal challenges with increasing dosages of TNBS, thereby combining two previously published protocols [5], [15]. This adapted protocol consistently resulted in mild to moderate colitis, reflected by significant growth retardation (p<0.005 ) (Figure 1A). Each rectal TNBS instillation induced transient loss of body weight of 5 to 15%. Average mortality was less than 15% and in particular associated with the first rectal challenge. At endpoint, colitis was evident from an increased colon weight/length ratio and increased thickening of the colon (Figure 1B). These macroscopic changes were associated with increased histology scores in the distal part of the colon (Figure 1C). Infiltration of mucosa and submucosa by CD4^+^ and CD8^+^ T cells, CD11b^+^ cells (Figure 2A), confirmed as macrophages by F4/80 staining (Figure 2B) was associated with mild to moderate damage of the mucosal architecture.

**Figure 3:**
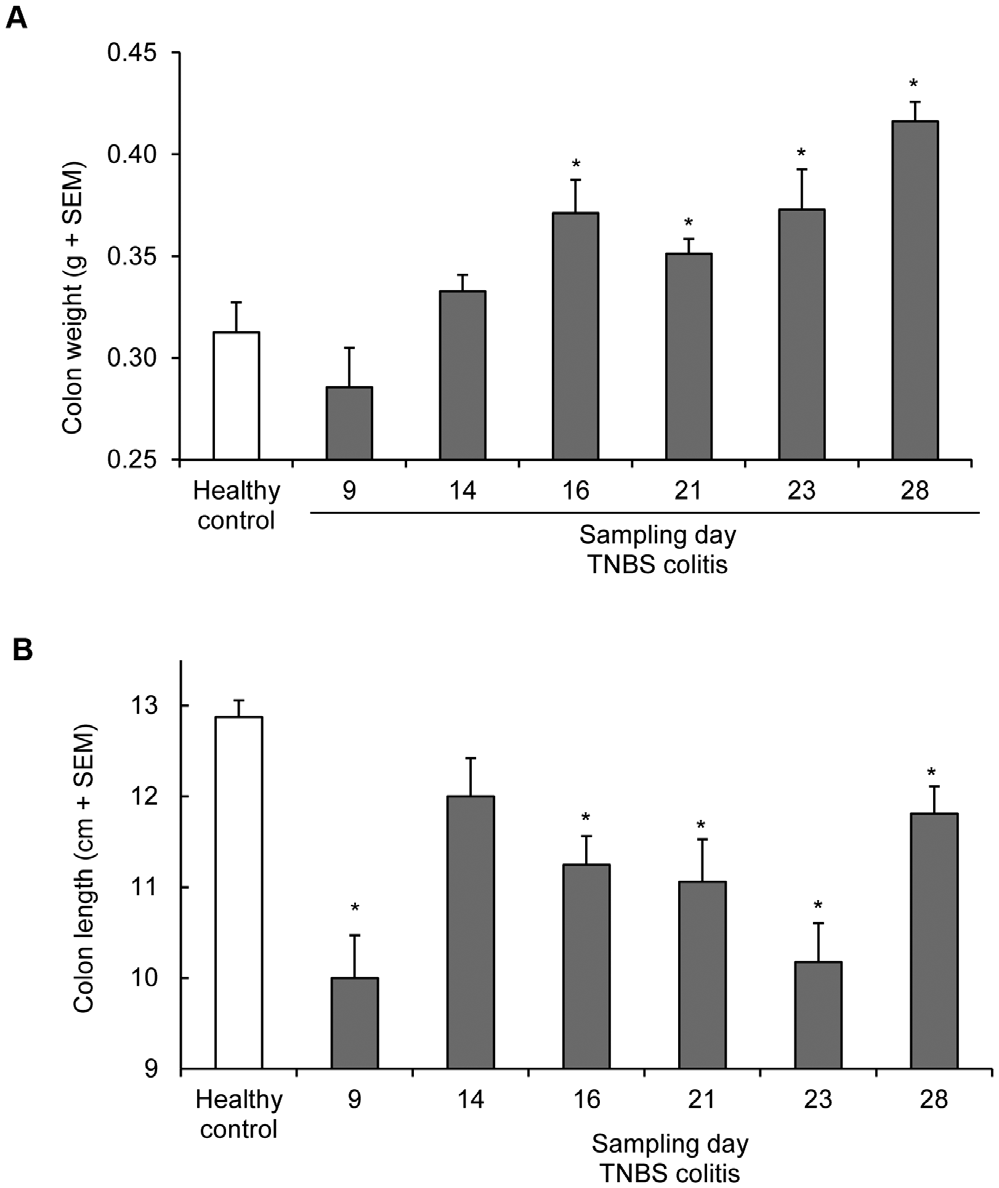
Disease characteristics of the recurrent TNBS model in time. Results of the time-course study in the recurrent TNBS colitis model. At each time-point indicated in Figure 8, 8 animals were sacrificed and colon length (**A**) and weight (**B**) was determined. Colitis is associated with a gradual increase in colon weight whereas the colon length is severely affected by each TNBS administration followed by a gradual relaxation.

All of these aspects were suppressed in mice treated by intrarectal administration of budesonide. Besides, budesonide treatment was associated with weight loss, typical for chronic exposure to corticosteroids in mice.

**Figure 4:**
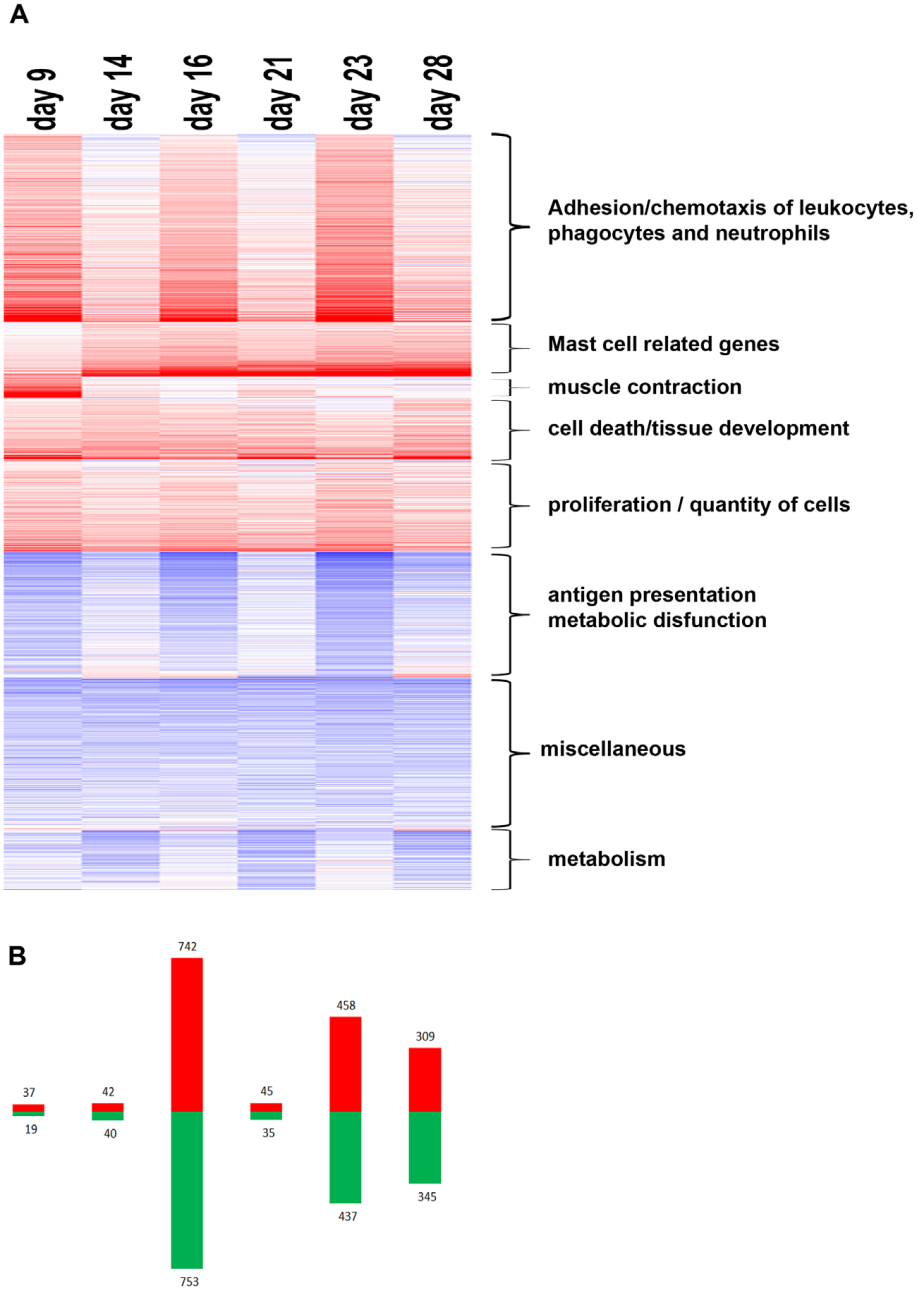
Transcriptome analysis in the recurrent TNBS-induced colitis model. To determine temporal patterns of gene expression levels that were affected by the repeated TNBS instillations, microarray analysis was performed on RNA isolated from colon tissue of colitic mice at day 9, 14, 16, 21, 23, and 28 (n = 5/group) (**A**) A heat-map of the genes with significantly modified gene expression revealed multiple clusters with different temporal expression patterns. (**B**) Bar graph indicates the number of up regulated (red) and down-regulated genes during colitis development at each time point. Genes were considered significant with a False discovery rate (FDR) p value <0.05. FDR was used to correct for multiple comparisons.

Multiplex analysis of 23 different cytokines and chemokines in serum collected at endpoint revealed significantly increased levels of IFN-γ, IL-1β, IL-17, MCP-1 and MIP-1β in mice with colitis, but not in mice treated with budesonide. Furthermore, budesonide suppressed IL-12p40 levels even compared to the serum concentrations in healthy control mice (Table 1).

**Figure 5:**
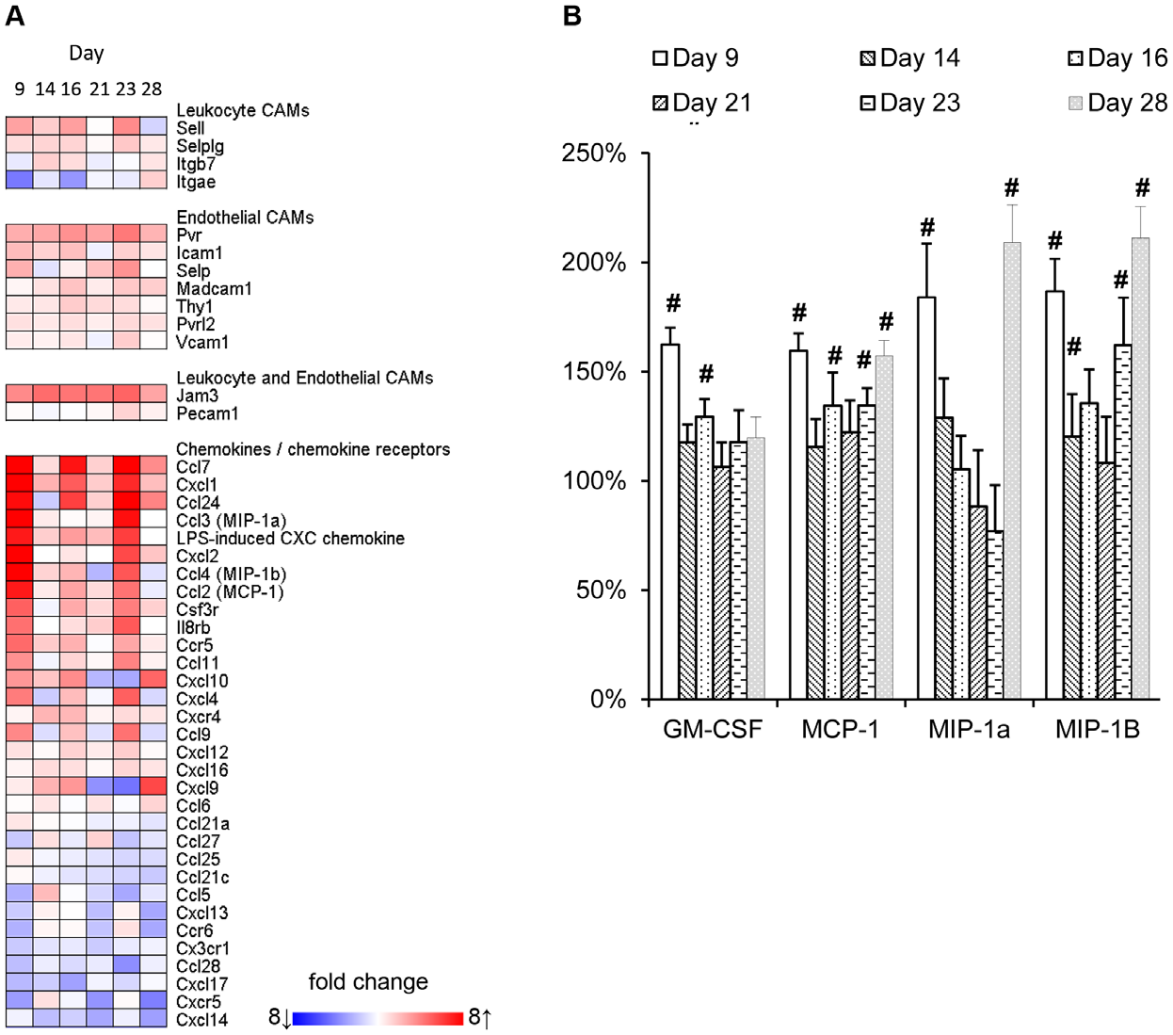
Effect of recurrent TNBS administrations on cell adhesion molecules and chemokines. (**A**) Local mRNA expression of cell adhesion molecules and chemokines was determined within the time-course microarray experiment. The size of the effect of TNBS-induced colitis on gene expression in comparison to healthy colons is indicated in a heatmap. These differences are based on mean expression levels of 5 individual animals per time-point. (**B**) Circulating serum chemokines were analyzed by multiplex technology. Relative serum concentrations of concentrations of GM-CSF, MCP-1 (CCL2), MIP-1α (CCL3), and MIP-1β (CCL4) are graphically depicted; significant differences are indicated by #.

**Table 1:** Relative circulating cytokine concentrations in TNBS-induced colitis^a^.

	Group								
Cytokine	Healthy (n = 14)			Vehicle (n = 19)			Budesonide(n = 15)		
Eotaxin	100%	±	5%	102%	±	9%	89%	±	10%
G-CSF	100%	±	10%	114%	±	9%	83%	±	8%^c^
GM-CSF	100%	±	4%	108%	±	5%	98%	±	7%
IFNγ	100%	±	10%	137%	±	12%^b^	93%	±	14%^c^
IL-10	100%	±	25%	194%	±	37%	54%	±	16%
IL-12p40	100%	±	8%	87%	±	7%	28%	±	4%^c^
IL-12p70	100%	±	15%	117%	±	19%	89%	±	11%
IL-13	100%	±	9%	133%	±	12%	82%	±	10%^c^
IL-17	100%	±	13%	153%	±	17%^b^	94%	±	12%^c^
IL-1α	100%	±	19%	93%	±	12%	87%	±	17%
IL-1β	100%	±	8%	135%	±	12%^b^	82%	±	9%^c^
IL-2	100%	±	11%	118%	±	12%	84%	±	12%
IL-3	100%	±	13%	137%	±	14%	104%	±	13%
IL-4	100%	±	15%	111%	±	12%	85%	±	14%
IL-5	100%	±	6%	111%	±	12%	72%	±	8%^c^
IL-6	100%	±	15%	122%	±	9%	82%	±	9%^c^
IL-9	100%	±	9%	118%	±	8%	84%	±	8%^c^
KC	100%	±	7%	107%	±	7%	81%	±	4%^c^
MCP-1	100%	±	9%	131%	±	9%^b^	86%	±	16%^c^
MIP-1α	100%	±	8%	95%	±	7%	96%	±	10%
MIP-1β	100%	±	6%	121%	±	6%^b^	100%	±	10%
Rantes	100%	±	6%	91%	±	5%	80%	±	7%
TNFα	100%	±	10%	106%	±	9%	116%	±	20%

a In two independent studies, serum concentrations of 23 cytokines were determined by multiplex technology on day 28. In Table S2 concentrations are presented as pg/ml ± SEM. In each study, values were normalized and expressed as a percentage of the mean concentration of the corresponding healthy control mice. Relative concentrations and shown as % ±SEM.

b significantly (p<0.05) different relative serum concentration from that in healthy mice.

c significantly (p<0.05) different relative serum concentration from that in vehicle treated mice.

Altogether, several inflammatory characteristics relevant to IBD are represented in this model and shown to be sensitive to corticosteroid treatment.

**Figure 6:**
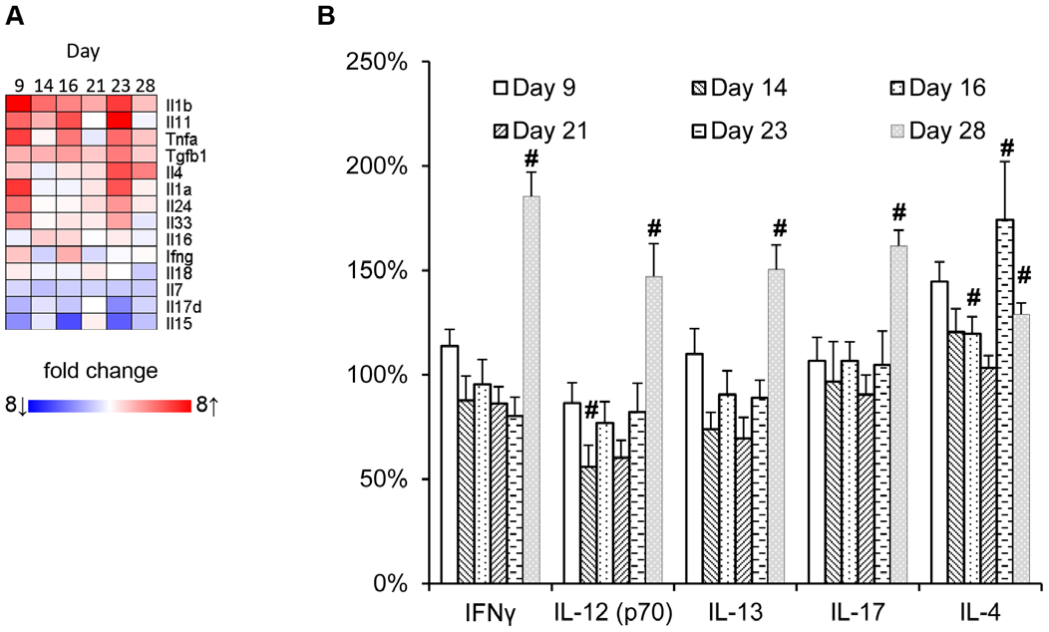
Effect of recurrent TNBS administrations on local and circulating cytokine profiles. (**A**) Local mRNA expression of cytokines was determined within the time-course microarray experiment. The size of the effect of TNBS-induced colitis on gene expression in comparison to healthy colons is indicated in a heatmap. These differences are based on mean expression levels of 5 individual animals per time-point. (**B**) Relative serum concentrations of Th1/Th2/Th17 hallmark cytokines at different time-points during the development of colitis. Concentrations of IFN-γ and IL-12p70, IL-4 and IL-13, and IL-17 are graphically depicted; significant differences are indicated by #.

### Alterations in Gene Expression during the Development of Recurrent TNBS Colitis

Because the adapted recurrent TNBS colitis model showed involvement of relevant pathological processes and sensitivity to treatment, we sought to gain more insight into processes involved in the early development of colitis in this model. Therefore, we evaluated gene expression in colons of mice sacrificed at different time-points, i.e. before the first and 2 or 7 days after each rectal TNBS instillation. Evaluation of colon macroscopy at each of these time-points showed that TNBS administration was associated with transient shortening of the colons, whereas colon weight increased gradually in time (Figure 3). Histological evaluation of the colons revealed marked areas of hemorrhages and complete destruction of crypt architecture two days after each TNBS instillation; this mucosal damage had partially recovered seven days after each instillation (data not shown).

**Figure 7:**
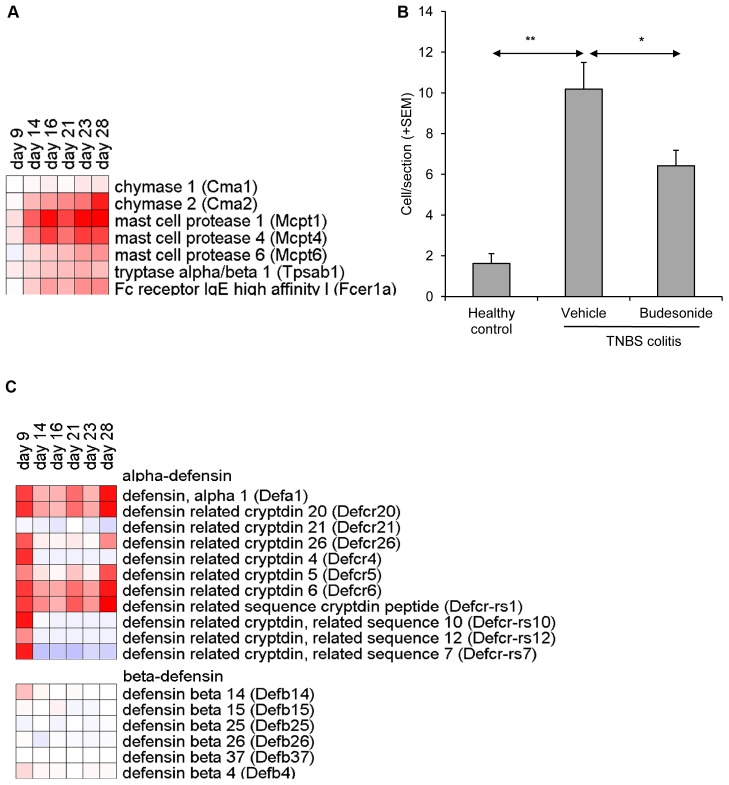
TNBS –induced colitis is associated with increased numbers of mast cells and α-defensins. (**A**) mRNA expression of selected mast cell specific genes in affected colon tissue (n = 5/group). Heatmap represents fold changes in TNBS colitis mice as compared to healthy control mice. (**B**) Quantification of toluidine blue positive cells confirmed TNBS induced up-regulation of mast cells. (**C**) mRNA expression of α- and β-defensin genes in affected colon tissue.

Genome wide expression profiling was performed on RNA isolated from distal colon tissue of five TNBS-treated mice at each selected time-point. Based on False Discovery Rate (FDR) correction, a total of 2074 genes of the 14285 transcripts that passed the filtering procedure, showed significantly different levels of mRNA at one or more time-points in comparison to mRNA from healthy mice. Differentially expressed genes that showed similar expression profiles are clustered in a heatmap (Figure 4A). The first TNBS challenge was associated with enhanced expression of a limited number of genes, whereas enhanced expression of a large number of genes occurred shortly after the second challenge on day 16 (Figure 4B). These patterns were largely normalized on day 21. Importantly, the third challenge resulted in extensive changes in gene expression on days 23 and 28. Forty-two % of the genes that showed increased expression on day 28 were also enhanced on day 23 and therefore not normalized. Furthermore, 27% of the genes differentially expressed on day 28, were not different from healthy on any of the earlier time-points, suggesting developing pathology upon multiple TNBS instillations.

**Figure 8:**
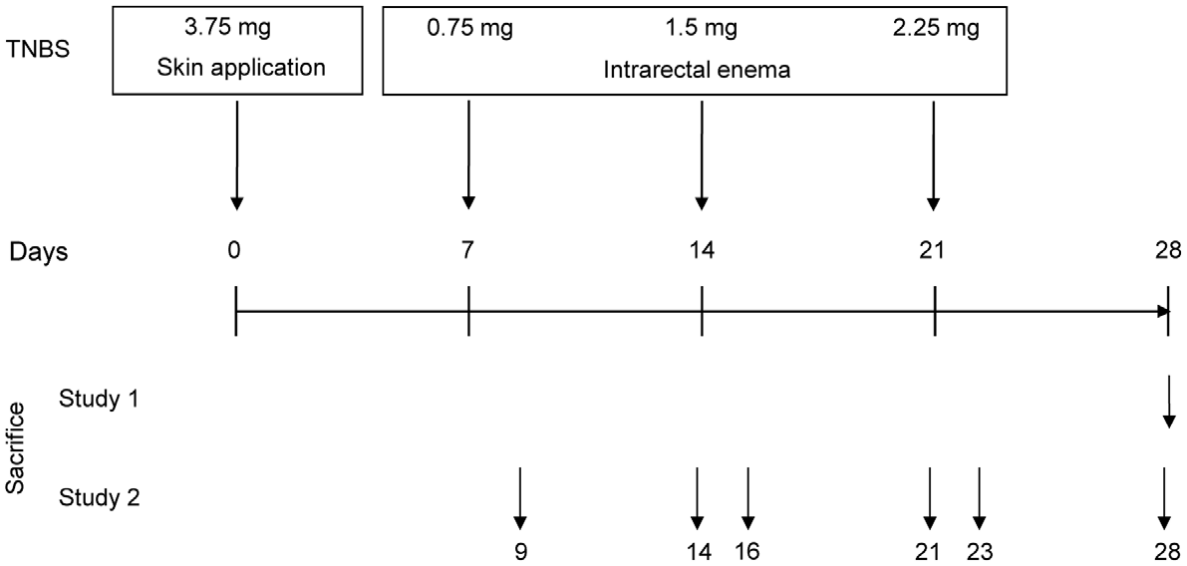
Schematic representation of the colitis induction protocol. Induction of colitis is achieved by dorsal skin application of TNBS in ethanol on day 0, followed by rectal TNBS/ethanol instillations on day 7, 14, and 21. The necropsy time-points for study 1, i.e. TNBS disease induction and corticosteroid treatment, and study 2, i.e. the time-course experiment, are presented in the upper part of the schematic. Severity of colitis induction was assessed by measurement of colon weight, length and thickness and by histopathological evaluation of the distal part of the colon. At each necropsy, serum was collected for cytokine profiling and colon tissue was sampled for RNA isolation.

To further elucidate the cellular processes associated with the induction of colitis by repeated TNBS instillations, we performed gene ontology analysis on the FDR filtered gene data set using MetaCore™ software. Enriched processes with more than 15 genes and p-values below 10−^5^ were considered to be significant (Table S1), which include general terms like “inflammation”, but also detailed underlying subcategories. Five major processes important for human pathogenesis of IBD were identified in this model, i.e. I) tissue morphogenesis, II) the response to wounding, III) immune/inflammatory responses, IV) cell adhesion and V) angiogenesis. Gene expression associated with immune/inflammatory responses was particularly evident 2 days after each TNBS challenge. Furthermore, several processes gradually developed in time, in particular processes associated with epithelial cell growth and tissue repair/wound healing (Table S1). Altogether, several processes found in human IBD are reflected in this recurrent TNBS model and these will be discussed more detailed in the next paragraphs. The key transcription factors involved in the processes underlying the development of colitis in this model were identified by MetaCore™ analysis. As shown in Table 2, Nuclear factor kappa-light-chain-enhancer of activated B cells (*NF-κB*), activator protein 1 (*AP-1*), CAMP responsive element binding protein 1 (*CREB1*), were central to the process at most time-points, in accordance with an inflammatory process. Networks involving Hepatocyte-Nuclear Factor-4α (*hnf4α*) were only observed on day 16 and 23, consistent with its role in the acute phase response.

**Table 2:** Master regulators in colitis development.

Transcription factor^a^	Central regulator of	Time-point^b^					
		9	14	16	21	23	28
SP1	Lipid metabolism, inflammation		++	++		++	++
HNF4α	Endodermal development			++		++	
c-myc	cell proliferation	+	+	+		+	+
CREB1	Inflammation			+		+	+
p53	Apoptosis, cell cycle	+	+	+		+	+
NF-κB	Inflammation	+	+	+	+	+	
AP1	Inflammation			+		+	+
C/EBP-β	Lipid metabolism, inflammation			+			+
ESR1	Metabolism, inflammation					+	+

a Transcription factors were identified in biological networks generated using MetaCore™ software. Transcription factors represented in this table were identified in significant networks containing a minimal of 8 nodes.

b Level of significance of the transcription factor network is classified based on z-scores: +, z-score between 50 and 100; ++, z-score above 100. No score means that the networks for these transcription factors were not identified at the time-point or had a z-score below 50.

### Chemokines, Cytokines and Cell-adhesion Molecules in Recurrent TNBS-colitis

Each of the repeated challenges with TNBS was followed by increased expression of genes encoding mediators of acute inflammation such TNF-α, IL-1β, CHI3L1, as well as calprotectin or calgranulin C (S100a8 -a9). These S100-proteins are relevant to the pathogenesis, disease activity, diagnosis, and therapeutic management of IBD [17]. Apart from this acute response towards TNBS instillations, the adaptive arm of the immune system appeared to be of importance from day 23 onwards as shown by gene ontology analysis in Metacore™. This analysis also revealed a role for cell adhesion molecules and chemokines. The KEGG list of 69 cell adhesion molecules (CAMs), chemokines and chemokine receptors, that was previously applied to illustrate an important role of these molecules in mucosal biopsies of UC and CD patients [18], was used to evaluate their expression during the development of recurrent TNBS-colitis. A heat-map of the molecules on this list that showed significantly altered expression at one or more time points is presented in Figure 5A. Several endothelial and leukocyte CAMs that were increased in patients with colonic IBD were also enhanced in the mouse model, e.g. CD62L, ICAM1, MADCAM1, SELE, THY1, JAM3, and PECAM1. Moreover, several chemokines involved in the recruitment of leukocytes, i.e., CCL2 (MCP-1), CCL3 (MIP-1α), CCL4 (MIP-1β), CCL7 (MCP-3), CCL9 (MIP-1γ), CCL24 (eotaxin-2), CXCL-1 (KC), and CXCL-2 (MIP-2α) were induced as well. Not all transcripts related to immune responses were up-regulated. Several genes encoding chemokines, including CCL21, and 25, and CXCL12, 14, and 15, showed gradually decreased expression during the development of colitis, indicative of a changing chemotactic profile during the progression of colon inflammation. Multiplex analysis on serum samples revealed that concentrations of GM-CSF, MCP-1, MIP-1α, and MIP-1β were significantly increased at one or more time-points during the experiment, although they did not reflect colon tissue gene expression profiles for these chemokines (Figure 5B).

As reported previously, repeated challenges with TNBS are associated with a changing cytokine profile in colon, suggesting for a shift from Th1 to a Th17 profile, and eventually Th2 cells with fibrosis [15]. In line with this study, IFN-γ gene expression was increased in our study, with a maximum on day 16, whereas IL-4 gene expression remained unchanged until the third TNBS challenge, after which a significant increase was observed (Figure 6A). However, mRNA expression profiles of cytokines considered significant for IBD or colitis, such IL-12p40, IL-23p19, IL-12p35, IL-17 did not differ from those in healthy tissue (data not shown).

To establish whether these IBD-related cytokines were reflected in the periphery, serum samples were subjected to cytokine analysis employing multiplex technology. A shown in Figure 6B, we did not obtain evidence for a biased cytokine profile at any time-point.

### Involvement of Mast Cells and α-defensins in TNBS-colitis

A small subset of genes showed gradually increasing expression levels in time. This cluster mainly comprised mast cell specific genes (Figure 7A), including mast cell protease 1, 4, and 6 (*mcpt1, 4, 6)*, mast cell chymase (*cma2,*), carboxypeptidase A3 (*cpa3*), tryptase α/β1 (*tpsab*), and high-affinity IgE Fc receptor (*fcer1a*). Gene expression was induced up to 233-fold (*mcpt1,* day 28). The highest expression levels of these genes were observed during the later stages of this model, i.e. on days 23 and 28. Immunohistochemical staining of FcεRI+ confirmed the presence of large numbers of mast cells in the lamina propria of affected colon tissue (data not shown). This was in line with increased numbers of mast cells stained by toluidine blue in affected colon tissue (Figure 7B). These results suggest a gradual increase of mast cells associated with the development of chronic colitis in this model.

Another set of genes that showed strongly affected expression levels encoded for antimicrobial peptides. Expression of many members of the α-defensin family was transiently increased on day 9, immediately following the first intrarectal TNBS instillation (Figure 7C). A subset of these defensins, including α-defensin 1 (*defa1*) and four α-defensin-related cryptdins (*defcr5*, *6*, *20* and *21* and *defcr-rs1*) also showed increased expression on day 28. These genes were induced up to 132-fold (*defcr-rs1*, day 28). This modulation was restricted to α-defensins, as the level β-defensin gene expression remained unchanged.

## Discussion

To gain insight into the pathogenesis of IBD, a variety of animal models reflecting different aspects of the disease are used [10], [11], [19]. Most IBD models result from exogenous manipulation; they can be categorized based on induction by chemicals, immune cell transfer, or gene targeting [19]. Models based on immune cell transfer, such as the CD45RB^Hi^ T-cell transfer model, and models based on gene targeting, such as the IL-10 knockout mouse model, present excellent models to study mechanisms involved in the pathogenesis IBD [20]. Nonetheless, these models have the disadvantage of lacking a fully functional immune system. These limitations do not apply for models of colitis induced by DSS or TNBS. However, these models usually have the disadvantage of being mediated by damage and being relatively insensitive to anti-inflammatory drugs. In this respect the recurrent TNBS colitis model is more promising. This model has been proposed because the induced pathology reflects the chronic and relapsing immune activation processes underlying human IBD resulting in late onset of fibrosis [13], [15]. In this report, we show results obtained in a modified version of this model, limiting the duration to first three weeks of colitis development. We show its sensitivity to immunosuppression by local corticosteroid treatment with budesonide which is a relevant compound in clinical treatment of patients with CD. Moreover, we recently showed that the model is sensitive to pretreatment with probiotic bacteria [16].

Our initial work to develop a model for colitis was largely inspired by the work of Neurath et al. who showed that TNBS-induced colitis is mediated by Th1 cells [21]. However, in our hands this model showed in SJL mice substantial mortality and variation, which was partly explained by the source of the animals and the type of chow diet (data not shown). Modifications to the protocol, as indicated by Wirtz et al [5], i.e. skin sensitization with TNBS prior to intrarectal challenges with TNBS, decreased the mortality rate in this model. Nevertheless, the limited therapeutic window of 2 days in the acute model prompted us to evaluate the characteristics of a recurrent TNBS-colitis model described by Fichtner-Feigl et al [15], allowing for prolonged treatment during a period of several weeks.

Our study further characterizes the processes underlying or associated with the early stage development of colitis in this model, i.e. a time frame of 3 weeks, by performing genome wide transcriptome analysis on colon tissue. This approach revealed that each intrarectal TNBS challenge induced a complex set of genes encoding molecules that are generally associated with an acute phase inflammatory response, such as TNFα, IL-6, calgranulins S100a8 and 9, and reg3γ. In addition, each intrarectal TNBS challenge was associated with an enhanced expression of genes encoding a set of chemokines; MCP-1 (CCL-2), MIP-1α (CCL3) and MIP-1β (CCL4), CCL7, CCL9, CCL11, CCL24, CXCL2 and CXCL4, showed a similar acute phase expression pattern, consistent with the influx of inflammatory cells. Moreover, serum concentrations of GM-CSF, MCP-1, MIP-1α, and MIP-1β were significantly increased in particular two days after each TNBS challenge. These data suggest that each TNBS challenge induces transient chemokine production in the colon, which likely drives the attraction of innate and adaptive immune cells to the site of TNBS-induced tissue damage. The involvement of these chemokines is relevant for human IBD since the majority is also elevated in tissues of UC and CD patients [22]–[24].

In addition, CXCL1 and CXCL2, which are chemokines with strong angiogenic effects, were strongly induced by the first intrarectal TNBS challenge and this was followed by the enrichment of a large cluster of genes encoding factors that are involved in angiogenesis, including FGF, endothelin-1, and VEGF-D. These data are suggestive for a link between the process of inflammation and angiogenesis in this model. Importantly, angiogenesis has been described as an important component in the pathogenesis and potential target for treatment of IBD [25].

Interestingly, the expression of a set of genes encoding chemokines involved in homeostatic processes, including CCL21, CCL25, CCL27 and CCL28, was gradually down-regulated in our TNBS colitis model. As these chemokines are involved in mucosal lymphoid structure development [26], our findings suggest that normal lymphatic tissue regeneration is affected in the model. This was substantiated by the observation that colitic tissue contained lower levels of transcripts associated with B cells (Cd19, Cxcr5, Tnfrsf13c, and Pou2af1) and intraepithelial lymphocytes [27] (IL-15, IL-7, and CD3γ).

Many genes that showed reduced expression upon TNBS colitis induction were related to metabolic processes. These main affected metabolic functions based on gene expression shifts comprise of changes in oxidative stress response processes, methylation, lipid biosynthesis, and small molecule metabolism. These observations reflect the strong connection between immune and metabolic response systems in the gut, as described Shulzhenko et al [28].

Mast cell influx is one of the key features of the model presented in this paper, as observed by microarray and histological examination. The transcriptomics data revealed progressively elevated mRNA levels of genes encoding mast cell specific products such as proteases 1, 4 and 6 (mcpt-1, -4, -6), carboxypeptidase 3 (cpa3), and high affinity Fc receptor for IgE (FcεRI). Mast cell infiltration was confirmed by increased numbers of toluidine blue positive mast cells as well as increases in FcεRI+ cells in the affected lamina propria. The attraction of mast cell progenitors from the blood depends on several factors, in particular local chemokine expression. These include, CCL2, CCXL4, CCL3, CCL24, and CCL11 [26], [29]. These chemokines were up- regulated during acute inflammation phases of the model and may therefore contribute to the temporal migration/recruitment of these cells to the inflamed mucosa. Interestingly, protective effects of probiotics in this model were associated with reduced expression of these chemokines [16]. Also in biopsies of CD and UC patients mucosal accumulation of mast cells has been observed [30]–[33]; however, the causative relationship between the development of IBD and numbers or activation state of mast cells has not been established. As mast cells are known to produce and release a plethora of mediators involved in pro-inflammatory as well as regulatory and active tissue repair processes [32], [34], [35], we hypothesize that they may exert differential effects depending on the stage of the inflammation processes.

The induction of colitis was associated with an increased expression of a cluster α-defensins together with the expression of other antimicrobial peptides, such as angiogenin 4 (ang4), regenerating islet derived antimicrobial peptides RegIIIγ and RegIV (reg3g and reg4, respectively), and secretory phospholipase A2 type IIA (pla2a2a/pla2g2a). Although all of these anti-microbial peptides have been described as products of Paneth cells in the small intestine, we have not been able to demonstrate the origin of these transcripts in the colon. Staining with Lendrum’s phloxine-tartrazine or immunohistochemical staining for lysozyme P did not confirm the presence of Paneth cells in the affected colon tissue (data not shown). It is tempting to speculate that these genes are indicative of the presence of metaplastic Paneth cells, which have been reported in both colonic CD and UC biopsies [36]–[38], whereas they are not found in healthy colon tissue [39], [40]. In patients these cells showed increased expression of human α-defensins DEFA5 and DEFA6 in the colon [22], [38], [41], [42]. Additional studies are needed to establish the origin of the antimicrobial peptide gene expression in affected colon tissue.

In conclusion, this recurrent TNBS-colitis model, adapted to comprise both transient acute inflammatory processes and gradually developing chronic inflammation, reflects several clinical and histopathological features of human IBD. The temporal gene expression profiles generated in this study revealed distinct processes involved in the onset and progression of disease in this model, and this knowledge may help to identify novel targets and therapeutic approaches for IBD.

## Materials and Methods

### Mouse Colitis Model

All experiments were performed with female BALB/c mice (8 wk old, 18–22 g) obtained from Janvier (St. Berthevin, France). Mice were conventionally housed under controlled temperature (22–24°C) and photoperiod (12 h light-dark cycle), and had free access to standard mouse chow (SSNIFF R/M-H, BioServices B.V., Uden, The Netherlands) and acidified tap water. Animal experiments were approved by the Institutional Animal Care and Use Committee of The Netherlands Organization for Applied Scientific Research (TNO), approval number DEC2982 and were in compliance with European Community specifications regarding the use of laboratory animals.

To induce colitis, mice were exposed to intracolonic administration of step-wise increasing doses of TNBS (Figure 8). Seven days prior to the first intracolonic TNBS administration, mice were sensitized by a single application of 3.75 mg of TNBS (Sigma-Aldrich, Zwijndrecht, The Netherlands) in 48% (v/v) ethanol on the shaved dorsal skin. On days 7, 14 and 21, mice were lightly anaesthesized with isoflurane and subsequently 0.75, 1.5 and 2.25 mg TNBS in 40% (v/v) ethanol was administered per rectum via a 2.0 mm tube (Unomedical A/S, Birkerød, Denmark) connected with a 1.0 ml syringe, which was advanced into the rectum for approximately 3 cm. Upon administration of 150 µl of TNBS solution, mice were held in a vertical position for 30 seconds to allow for equal distribution.

### Study Design

In this report, the results of 2 experiments (Figure 8) are discussed in detail. In study 1, induction of colitis was performed as described above and at day 28 all animals were sacrificed for assessment of all relevant parameters. In this study, local budesonide (3 mg/kg) treatment was included as a positive treatment control. Budesonide was administered 3× per week starting one day prior to the first rectal TNBS administration, i.e. day 6. Budesonide (Sigma-Aldrich, Zwijndrecht, The Netherlands) was prepared in PBS containing 2.5% (v/v) Tween-80 and 2.5% (v/v) ethanol. In study 2, we performed a time-course study to evaluate the development of colitis by macroscopy, cytokine profiling and gene expression profiling. In this study, one group of animals (n = 8) was included for each time-point in the study. The selected time-points 2 and 7 days after each TNBS administration, 8 mice were sacrificed, as indicated in Figure 8.

### Colon Macroscopy

Severity of colitis was assessed by macroscopical analysis directly upon sacrifice. After colon length was measured, feces were removed by gentle pressure with forceps. Subsequently, colon weight was measured. Finally, colon thickness was measured with a digital caliper at 1, 2, and 3 cm from the anus.

### Histological Assessment of Colitis

Following measurement of macroscopy parameters, colons were dissected and fragments were fixed in 4% buffered formalin and embedded in paraffin. Sections of 5 µm were stained by hematoxylin-eosin-saffron. Inflammation was scored in a blinded manner for 3 non-sequential sections per colon fragment at a 400× magnification, according to a semi quantitative (0–4) scoring system, based on number of infiltrating cells and mucosal damage. Mast cells were evaluated after staining with toluidine blue [43] at a 400× magnification.

### Immunohistochemical Analysis of Cellular Infiltrates

Dissected colon fragments were immediately immersed in Tissue Tek OCT compound (Sakura Finetel, Torrance, CA, USA) and cryopreserved in liquid nitrogen. Immunohistochemistry was performed on 6 µm sections with primary antibodies specific for CD4 (L3T4.), CD8 (53–6.7), CD11b (M1/70), and F4/80 (CI-A3-1all obtained from BD Biosciences (San Diego, CA, USA). After incubation, biotinylated antibodies were detected by incubation with streptavidin-HRP (Vector Laboratories, Burlingame, CA, USA) using 3-amino-9-ethylcarbozole (Sigma) as a substrate. Immunopositive cells were counted at a 400× magnification and normalized against the colon tissue surface area. For immunofluorescence, primary antibodies and according Texas red and fluorescein labeled secondary antibodies were applied. Slides were covered with Vectashield (H1000) mounting medium with DAPI Vector).

### Cytokine and Chemokine Quantification in Serum

Cytokine concentrations in serum obtained at each sacrifice were determined by Multiplex analyses. For this purpose, serum was diluted and assayed with the Bio-Plex Pro 23-Plex Panel, for IL-1α, -1β, -2, -3, -4, -5, -6, -9, -10, -12(p40), -12(p70), -13, -17, IFN-γ, TNF-α, RANTES, MIP-1α and -1β, MCP-1, KC, G-CSF, GM-CSF, and eotaxin protein (Bio-Rad Laboratories, Hercules, CA, USA). The beads were read on a LiquiChip 200, (Qiagen, Hombrechtikon, Switzerland), and data were analyzed by the five parameter curve fitting in Luminex100 IS Software. Cytokine concentrations were corrected for the dilution factor and presented in pg/ml or as relative concentrations to the average concentration in healthy mice.

### Transcriptome Analysis

Total RNA from frozen intestinal tissue was isolated using TRIzol reagent (Invitrogen, Breda, The Netherlands) according to the manufacturer’s instructions. RNA was treated with DNAse and purified using Nucleospin RNAII Total RNA Isolation kit (Macherey-Nagel, Düren, Germany), according to manufacturer’s protocol.

The quality control of RNA samples, RNA labeling and hybridization were performed at ServiceXS (Leiden, The Netherlands). RNA concentration was assessed using a Nanodrop ND-1000 spectrophotometer (Nanodrop Technologies, Wilmington, DE, U.S.A). The RNA quality and integrity was determined using Lab-on-Chip analysis on an Agilent 2100 Bioanalyzer (Agilent Technologies, Inc., Santa Clara, CA, U.S.A.). The RNA integrity numbers (RIN) of all RNA samples had values above 7.3. Biotinylated cRNA was prepared using the Illumina TotalPrep RNA Amplification Kit (Ambion, Inc., Austin, TX, U.S.A.) according to the manufacturer’s specifications starting with 500 ng total RNA. Per sample, 750 ng of cRNA was used to hybridize to the Sentrix MouseRef-8 BeadChips (Illumina, Inc., San Diego, CA, U.S.A.). Each BeadChip contains eight arrays and each of the arrays harbors 25697 probes. Hybridization and washing were performed according to the Illumina standard assay procedure. Scanning was performed on the Illumina iScanner (Illumina, Inc., San Diego, CA, U.S.A.). Image analysis and extraction of raw and background subtracted expression data were performed with Illumina Beadstudio v3 Gene Expression software using default settings. The data discussed in this publication have been deposited in NCBI’s Gene Expression Omnibus [44] and are accessible through GEO Series accession number GSE35609 (http://www.ncbi.nlm.nih.gov/geo/query/acc.cgi?acc=GSE35609). GeneSpring GX 11.0 was used for quantile normalization of the probe-level, background subtracted expression values. After the normalization, unexpressed probes were removed from the further analyses. All expression values below 5 (2.322 on log2 scale) were floored to 5. Differentially expressed probes were identified using the limma package of the R/Bioconductor project, applying linear models and moderated t-statistics that implement empirical Bayes regularization of standard errors [45]. The statistical analyses were performed through The Remote Analysis Computation for gene Expression data (RACE) suite at http://race.unil.ch [46]. False Discovery Rate (FDR) corrected p-values of 0.05 was used as threshold for significance of the differential expression. Fold changes relative to healthy control mice are presented. GEO Processes were identified using Metacore Software V6.11.

RT-qPCR for a set of genes (CCL2, CCL11, CCL24, cldn-1, cldn-4, cldn-11) showed a high correlation with data obtained by microarrays, and thereby confirmed the relative gene expression presented in the figures (data not shown).

## Supporting Information

Table S1 **The enrichment of GO biological process terms on day 9, 14, 16, 21, 23 and 28 in the recurrent TNBS colitis model.**
(XLSX)

Table S2 **Serum cytokines and chemokines in TNBS-induced colitis.**
(DOC)

## References

[1] Engel MA, Neurath MF (2010) New pathophysiological insights and modern treatment of IBD. Journal of Gastroenterology 45: 571–583. 20213337 10.1007/s00535-010-0219-3

[2] Xavier RJ, Podolsky DK (2007) Unravelling the pathogenesis of inflammatory bowel disease. Nature 448: 427–434. 17653185 10.1038/nature06005

[3] Kozuch PL, Hanauer SB (2008) Treatment of inflammatory bowel disease: A review of medical therapy. World Journal of Gastroenterology : WJG 14: 354–377. 18200659 10.3748/wjg.14.354 PMC2679125

[4] Kawada M, Arihiro A, Mizoguchi E (2007) Insights from advances in research of chemically induced experimental models of human inflammatory bowel disease. World Journal of Gastroenterology : WJG 13: 5581–5593. 17948932 10.3748/wjg.v13.i42.5581 PMC4172737

[5] Wirtz S, Neufert C, Weigmann B, Neurath MF (2007) Chemically induced mouse models of intestinal inflammation. Nature Protocols 2: 541–546. 17406617 10.1038/nprot.2007.41

[6] Neurath M, Fuss I, Strober W (2000) TNBS-colitis. International Reviews of Immunology 19: 51–62. 10723677 10.3109/08830180009048389

[7] Paiotti AP, Miszputen SJ, Oshima CT, Artigiani Neto R, Ribeiro DA, et al (2011) Etanercept attenuates TNBS-induced experimental colitis: Role of TNF-alpha expression. Journal of Molecular Histology 42: 443–450. 21863329 10.1007/s10735-011-9349-z

[8] Shen C, de Hertogh G, Bullens DM, Van Assche G, Geboes K, et al (2007) Remission-inducing effect of anti-TNF monoclonal antibody in TNBS colitis: Mechanisms beyond neutralization? Inflammatory Bowel Diseases 13: 308–316. 17206708 10.1002/ibd.20005

[9] Mencarelli A, Renga B, Palladino G, Distrutti E, Fiorucci S (2009) The plant sterol guggulsterone attenuates inflammation and immune dysfunction in murine models of inflammatory bowel disease. Biochemical Pharmacology 78: 1214–1223. 19555671 10.1016/j.bcp.2009.06.026

[10] Elson CO, Cong Y, Lorenz R, Weaver CT (2004) New developments in experimental models of inflammatory bowel disease. Current Opinion in Gastroenterology 20: 360–367. 15703665 10.1097/00001574-200407000-00010

[11] Strober W, Fuss IJ (2006) Experimental models of mucosal inflammation. Advances in Experimental Medicine and Biology 579: 55–97. 16620012 10.1007/0-387-33778-4_5

[12] Fichtner-Feigl S, Fuss IJ, Preiss JC, Strober W, Kitani A (2005) Treatment of murine Th1- and Th2-mediated inflammatory bowel disease with NF-kappa B decoy oligonucleotides. The Journal of Clinical Investigation 115: 3057–3071. 16239967 10.1172/JCI24792 PMC1257534

[13] Lawrance IC, Wu F, Leite AZ, Willis J, West GA, et al (2003) A murine model of chronic inflammation-induced intestinal fibrosis down-regulated by antisense NF-kappa B. Gastroenterology. 125: 1750–1761. 10.1053/j.gastro.2003.08.027 14724828

[14] Fichtner-Feigl S, Strober W, Geissler EK, Schlitt HJ (2008) Cytokines mediating the induction of chronic colitis and colitis-associated fibrosis. Mucosal Immunology 1 Suppl 1 S24–7. 19079223 10.1038/mi.2008.41 PMC3673699

[15] Fichtner-Feigl S, Fuss IJ, Young CA, Watanabe T, Geissler EK, et al (2007) Induction of IL-13 triggers TGF-beta1-dependent tissue fibrosis in chronic 2,4,6-trinitrobenzene sulfonic acid colitis. Journal of Immunology (Baltimore, Md. : 1950) 178: 5859–5870. 10.4049/jimmunol.178.9.5859 17442970

[16] Mariman R, Kremer B, van Erk M, Lagerweij T, Koning F, et al (2012) Gene expression profiling identifies mechanisms of protection to recurrent trinitrobenzene sulfonic acid colitis mediated by probiotics. Inflammatory Bowel Diseases 18: 1424–1433. 22162025 10.1002/ibd.22849

[17] Manolakis AC, Kapsoritakis AN, Tiaka EK, Potamianos SP (2011) Calprotectin, calgranulin C, and other members of the s100 protein family in inflammatory bowel disease. Digestive Diseases and Sciences 56: 1601–1611. 21203903 10.1007/s10620-010-1494-9

[18] Arijs I, De Hertogh G, Machiels K, Van Steen K, Lemaire K, et al (2011) Mucosal gene expression of cell adhesion molecules, chemokines, and chemokine receptors in patients with inflammatory bowel disease before and after infliximab treatment. The American Journal of Gastroenterology 106: 748–761. 21326222 10.1038/ajg.2011.27

[19] Wirtz S, Neurath MF (2007) Mouse models of inflammatory bowel disease. Advanced Drug Delivery Reviews 59: 1073–1083. 17825455 10.1016/j.addr.2007.07.003

[20] Strober W (2008) Why study animal models of IBD? Inflammatory Bowel Diseases 14 Suppl 2 S129–31. 18816654 10.1002/ibd.20667

[21] Neurath MF, Fuss I, Kelsall BL, Stuber E, Strober W (1995) Antibodies to interleukin 12 abrogate established experimental colitis in mice. The Journal of Experimental Medicine 182: 1281–1290. 7595199 10.1084/jem.182.5.1281 PMC2192205

[22] Noble CL, Abbas AR, Cornelius J, Lees CW, Ho GT, et al (2008) Regional variation in gene expression in the healthy colon is dysregulated in ulcerative colitis. Gut 57: 1398–1405. 18523026 10.1136/gut.2008.148395

[23] Noble CL, Abbas AR, Lees CW, Cornelius J, Toy K, et al (2010) Characterization of intestinal gene expression profiles in crohn’s disease by genome-wide microarray analysis. Inflammatory Bowel Diseases 16: 1717–1728. 20848455 10.1002/ibd.21263

[24] Gijsbers K, Geboes K, Van Damme J (2006) Chemokines in gastrointestinal disorders. Current Drug Targets 7: 47–64. 16454699 10.2174/138945006775270222

[25] Danese S, Sans M, de la Motte C, Graziani C, West G, et al (2006) Angiogenesis as a novel component of inflammatory bowel disease pathogenesis. Gastroenterology 130: 2060–2073. 16762629 10.1053/j.gastro.2006.03.054

[26] Zimmerman NP, Vongsa RA, Wendt MK, Dwinell MB (2008) Chemokines and chemokine receptors in mucosal homeostasis at the intestinal epithelial barrier in inflammatory bowel disease. Inflammatory Bowel Diseases 14: 1000–1011. 18452220 10.1002/ibd.20480 PMC4114077

[27] Porter BO, Malek TR (2000) Thymic and intestinal intraepithelial T lymphocyte development are each regulated by the gammac-dependent cytokines IL-2, IL-7, and IL-15. Seminars in Immunology 12: 465–474. 11085179 10.1006/smim.2000.0264

[28] Shulzhenko N, Morgun A, Hsiao W, Battle M, Yao M, et al (2011) Crosstalk between B lymphocytes, microbiota and the intestinal epithelium governs immunity versus metabolism in the gut. Nature Medicine 17: 1585–1593. 10.1038/nm.2505 PMC3902046 22101768

[29] Hallgren J, Gurish MF (2011) Mast cell progenitor trafficking and maturation. Advances in Experimental Medicine and Biology 716: 14–28. 21713649 10.1007/978-1-4419-9533-9_2 PMC3554263

[30] Stenton GR, Vliagoftis H, Befus AD (1998) Role of intestinal mast cells in modulating gastrointestinal pathophysiology. Annals of Allergy, Asthma & Immunology : Official Publication of the American College of Allergy, Asthma, & Immunology 81: 1–11; quiz 12–5. 10.1016/S1081-1206(10)63105-5 9690568

[31] Raithel M, Matek M, Baenkler HW, Jorde W, Hahn EG (1995) Mucosal histamine content and histamine secretion in crohn’s disease, ulcerative colitis and allergic enteropathy. International Archives of Allergy and Immunology 108: 127–133. 7549499 10.1159/000237129

[32] He SH (2004) Key role of mast cells and their major secretory products in inflammatory bowel disease. World Journal of Gastroenterology : WJG 10: 309–318. 14760748 10.3748/wjg.v10.i3.309 PMC4724914

[33] Andoh A, Deguchi Y, Inatomi O, Yagi Y, Bamba S, et al (2006) Immunohistochemical study of chymase-positive mast cells in inflammatory bowel disease. Oncology Reports 16: 103–107. 16786130

[34] Rijnierse A, Nijkamp FP, Kraneveld AD (2007) Mast cells and nerves tickle in the tummy: Implications for inflammatory bowel disease and irritable bowel syndrome. Pharmacology & Therapeutics 116: 207–235. 17719089 10.1016/j.pharmthera.2007.06.008

[35] Theoharides TC, Alysandratos KD, Angelidou A, Delivanis DA, Sismanopoulos N, et al (2012) Mast cells and inflammation. Biochimica Et Biophysica Acta 1822: 21–33. 21185371 10.1016/j.bbadis.2010.12.014 PMC3318920

[36] Cunliffe RN, Rose FR, Keyte J, Abberley L, Chan WC, et al (2001) Human defensin 5 is stored in precursor form in normal paneth cells and is expressed by some villous epithelial cells and by metaplastic paneth cells in the colon in inflammatory bowel disease. Gut 48: 176–185. 11156637 10.1136/gut.48.2.176 PMC1728187

[37] Fahlgren A, Hammarstrom S, Danielsson A, Hammarstrom ML (2003) Increased expression of antimicrobial peptides and lysozyme in colonic epithelial cells of patients with ulcerative colitis. Clinical and Experimental Immunology 131: 90–101. 12519391 10.1046/j.1365-2249.2003.02035.x PMC1808590

[38] Perminow G, Beisner J, Koslowski M, Lyckander LG, Stange E, et al (2010) Defective paneth cell-mediated host defense in pediatric ileal crohn’s disease. The American Journal of Gastroenterology 105: 452–459. 19904243 10.1038/ajg.2009.643

[39] Sommers SC (1966) Mast cells and paneth cells in ulcerative colitis. Gastroenterology 51: 841–850. 5332241

[40] Boulton RA, Usselmann B, Mohammed I, Jankowski J (2003) Barrett’s esophagus: Environmental influences in the progression of dysplasia. World Journal of Surgery 27: 1014–1017. 12879287 10.1007/s00268-003-7054-0

[41] Wehkamp J, Stange EF (2006) Paneth cells and the innate immune response. Current Opinion in Gastroenterology 22: 644–650. 17053443 10.1097/01.mog.0000245541.95408.86

[42] Zilbauer M, Jenke A, Wenzel G, Goedde D, Postberg J, et al (2011) Intestinal alpha-defensin expression in pediatric inflammatory bowel disease. Inflammatory Bowel Diseases 17: 2076–2086. 21910169 10.1002/ibd.21577

[43] Strobel S, Miller HR, Ferguson A (1981) Human intestinal mucosal mast cells: Evaluation of fixation and staining techniques. Journal of Clinical Pathology 34: 851–858. 6168659 10.1136/jcp.34.8.851 PMC493957

[44] Edgar R, Domrachev M, Lash AE (2002) Gene expression omnibus: NCBI gene expression and hybridization array data repository. Nucleic Acids Research 30: 207–210. 11752295 10.1093/nar/30.1.207 PMC99122

[45] Smyth GK (2004) Linear models and empirical bayes methods for assessing differential expression in microarray experiments. Statistical Applications in Genetics and Molecular Biology 3: Article3. 16646809 10.2202/1544-6115.1027

[46] Psarros M, Heber S, Sick M, Thoppae G, Harshman K, et al (2005) RACE: Remote analysis computation for gene expression data. Nucleic Acids Research 33: W638–43. 15980552 10.1093/nar/gki490 PMC1160250

