# Supplementary material for: Temporal Colonic Gene Expression Profiling in the Recurrent Colitis Model Identifies Early and Chronic Inflammatory Processes
Source: PLoS One. 2012 Nov 30;7(11):e50388. doi: 10.1371/journal.pone.0050388 (PMC3511545; doi:10.1371/journal.pone.0050388)
Supplement: Table S2 — Serum cytokines and chemokines in TNBS-induced colitis. (DOC) [file pone.0050388.s002.doc]

**Supporting Table 2.**

| **Serum cytokines and chemokines in TNBS-induced colitis** | | | | | | | | | | | | | | | | | |
| --- | --- | --- | --- | --- | --- | --- | --- | --- | --- | --- | --- | --- | --- | --- | --- | --- | --- |
|  | **Study A^a^** | | | **Study B^a^** | | | **Relative concentration as % of healthy control** ^b^ | | | | | | | | | | |
|  |  |  |  |  |  |  |  | | |  |  | | |  |  | | |
|  | healthy | vehicle | budesonide | healthy | vehicle | budesonide | healthy | | |  | vehicle | | |  | budesonide | | |
| **Cytokine** | (n=6) | (n=10) | (n=6) | (n=8) | (n=9) | (n=9) |  |  |  |  |  |  |  |  |  |  |  |
| Eotaxin | 4400 ± 350 | 4664 ± 456 | 4961 ± 560 | 11273 ± 820 | 12084± 1983 | 8339±1478 | 100 | ± | 5 |  | 102 | ± | 9 |  | 89 | ± | 10 |
| G-CSF | 38 ± 8 | 42 ± 7 | 36 ± 6 | 543 ± 44 | 581 ± 39 | 403 ± 39 | 100 | ± | 10 |  | 114 | ± | 9 |  | 83 | ± | 8^d^ |
| GM-CSF | 185 ± 17 | 214 ± 14 | 193 ± 25 | 1461 ± 58 | 1532 ± 99 | 1382 ± 104 | 100 | ± | 4 |  | 108 | ± | 5 |  | 98 | ± | 7 |
| IFNγ | 530 ± 98 | 763 ± 58 | 654 ± 144 | 743 ± 96 | 996 ± 163 | 542 ± 88 | 100 | ± | 10 |  | 137 | ± | 12^c^ |  | 93 | ± | 14^d^ |
| IL-10 | 63 ± 21 | 196 ± 32 | 67 ± 14 | 179 ± 66 | 163 ± 43 | 45 ± 25 | 100 | ± | 25 |  | 194 | ± | 37 |  | 54 | ± | 16 |
| IL-12p40 | 545 ± 80 | 458 ± 71 | 61 ± 6 | 2302 ± 199 | 1900 ± 143 | 884 ± 88 | 100 | ± | 8 |  | 87 | ± | 7 |  | 28 | ± | 4^d^ |
| IL-12p70 | 46 ± 15 | 58 ± 16 | 76 ± 42 | 851 ± 101 | 970 ± 143 | 845 ± 114 | 100 | ± | 15 |  | 117 | ± | 19 |  | 89 | ± | 11 |
| IL-13 | 363 ± 66 | 526 ± 91 | 294 ± 71 | 3692 ± 307 | 5167 ± 638 | 3060 ± 404 | 100 | ± | 9 |  | 133 | ± | 12 |  | 82 | ± | 10^d^ |
| IL-17 | 77 ± 23 | 149 ± 21 | 74 ± 22 | 873 ± 86 | 1085 ± 157 | 805 ± 85 | 100 | ± | 13 |  | 153 | ± | 17^c^ |  | 94 | ± | 12^d^ |
| IL-1α | 237 ± 37 | 273 ± 19 | 246 ± 50 | 128 ± 41 | 86 ± 26 | 97 ± 31 | 100 | ± | 19 |  | 93 | ± | 12 |  | 87 | ± | 17 |
| IL-1β | 1005 ± 155 | 1499 ± 173 | 776 ± 186 | 2979 ± 298 | 3875 ± 551 | 2547 ± 313 | 100 | ± | 8 |  | 135 | ± | 12^c^ |  | 82 | ± | 9^d^ |
| IL-2 | 25 ± 6 | 30 ± 5 | 24 ± 6 | 574 ± 61 | 680 ± 81 | 435 ± 62 | 100 | ± | 11 |  | 118 | ± | 12 |  | 84 | ± | 12 |
| IL-3 | 11 ± 2 | 20 ± 5 | 14 ± 3 | 47 ± 8 | 66 ± 12 | 42 ± 5 | 100 | ± | 13 |  | 137 | ± | 14 |  | 104 | ± | 13 |
| IL-4 | 25 ± 4 | 31 ± 4 | 28 ± 6 | 94 ± 22 | 105 ± 22 | 64 ± 16 | 100 | ± | 15 |  | 111 | ± | 12 |  | 85 | ± | 14 |
| IL-5 | 80 ± 9 | 82 ± 17 | 49 ± 9 | 415 ± 31 | 573 ± 79 | 324 ± 43 | 100 | ± | 6 |  | 111 | ± | 12 |  | 72 | ± | 8^d^ |
| IL-6 | 52 ± 10 | 58 ± 8 | 40 ± 12 | 66 ± 15 | 80 ± 9 | 56 ± 4 | 100 | ± | 15 |  | 122 | ± | 9 |  | 82 | ± | 9^d^ |
| IL-9 | 193 ± 38 | 211 ± 19 | 156 ± 34 | 2392 ± 153 | 3012 ± 326 | 2066 ± 152 | 100 | ± | 9 |  | 118 | ± | 8 |  | 84 | ± | 8^d^ |
| KC | 213 ± 32 | 249 ± 25 | 171 ± 18 | 483 ± 26 | 472 ± 19 | 396 ± 23 | 100 | ± | 7 |  | 107 | ± | 7 |  | 81 | ± | 4^d^ |
| MCP-1 | 561 ± 99 | 878 ± 92 | 740 ± 144 | 2864 ± 261 | 3384 ± 411 | 1584 ± 346 | 100 | ± | 9 |  | 131 | ± | 9^c^ |  | 86 | ± | 16^d^ |
| MIP-1α | 1593 ± 177 | 1812 ± 167 | 1982 ± 190 | 5041 ± 554 | 4089 ± 370 | 3923 ± 512 | 100 | ± | 8 |  | 95 | ± | 7 |  | 96 | ± | 10 |
| MIP-1β | 130 ± 20 | 161 ± 9 | 156 ± 26 | 1204 ± 54 | 1409 ± 133 | 1041 ± 103 | 100 | ± | 6 |  | 121 | ± | 6^c^ |  | 100 | ± | 10 |
| Rantes | 478 ± 50 | 492 ± 69 | 453 ± 66 | 187 ± 15 | 171 ± 17 | 131 ± 13 | 100 | ± | 6 |  | 91 | ± | 5 |  | 80 | ± | 7 |
| TNFα | 2956 ± 671 | 1894 ± 333 | 4584 ± 1425 | 2609 ± 130 | 3263 ± 386 | 2334 ± 168 | 100 | ± | 10 |  | 106 | ± | 9 |  | 116 | ± | 20 |

^a^, In two independent studies, serum concentrations of 23 cytokines were determined by multiplex technology on day 28. Concentrations are presented as pg/ml ± SEM .

^b^ In each study, values were normalized and expressed as a percentage of the mean concentration of the corresponding healthy control mice. Relative concentrations and shown as % ±SEM.

^c^, significantly (p<0.05) different relative serum concentration from that in healthy mice

^d^, significantly (p<0.05) different relative serum concentration from that in vehicle treated mice.
